# Evaluation of New Morphometric Parameters of Neoangiogenesis in Human Colorectal Cancer Using Confocal Laser Endomicroscopy (CLE) and Targeted Panendothelial Markers

**DOI:** 10.1371/journal.pone.0091084

**Published:** 2014-03-10

**Authors:** Adriana Ciocâlteu, Adrian Săftoiu, Tatiana Cârţână, Lucian Gheorghe Gruionu, Daniel Pirici, Corneliu Cristian Georgescu, Claudia-Valentina Georgescu, Dan Ionuţ Gheonea, Gabriel Gruionu

**Affiliations:** 1 Research Center of Gastroenterology and Hepatology, University of Medicine and Pharmacy of Craiova, Craiova, Romania; 2 Department of Mechanical Engineering, University of Craiova, Craiova, Romania; 3 Department of Histology, University of Medicine and Pharmacy of Craiova, Craiova, Romania; 4 Department of Pharmacology, University of Medicine and Pharmacy of Craiova, Craiova, Romania; 5 Department of Anesthesiology and Intensive Care, Emergency County Hospital, Craiova, Romania; 6 Department of Pathology, Emergency County Hospital, Craiova, Romania; 7 Edwin L. Steele Laboratory of Tumor Biology, Department of Radiation Oncology, Massachusetts General Hospital, Harvard Medical School, Boston, Massachusetts, United States of America; University of Bari Medical School, Italy

## Abstract

The tumor microcirculation is characterized by an abnormal vascular network with dilated, tortuous and saccular vessels. Therefore, imaging the tumor vasculature and determining its morphometric characteristics represent a critical goal for optimizing the cancer treatment that targets the blood vessels (i.e. antiangiogenesis therapy). The aim of this study was to evaluate new vascular morphometric parameters in colorectal cancer, difficult to achieve through conventional immunohistochemistry, by using the confocal laser endomicroscopy method. Fresh biopsies from tumor and normal tissue were collected during colonoscopy from five patients with T3 colorectal carcinoma without metastasis and were marked with fluorescently labeled anti-CD31 antibodies. A series of optical slices spanning 250 µm inside the tissue were immediately collected for each sample using a confocal laser endomicroscope. All measurements were expressed as the mean ± standard error. The mean diameter of tumor vessels was significantly larger than the normal vessels (9.46±0.4 µm vs. 7.60±0.3 µm, p = 0.0166). The vessel density was also significantly higher in the cancer vs. normal tissue samples (5541.05±262.81 vs. 3755.79±194.96 vessels/mm^3^, p = 0.0006). These results were confirmed by immunohistochemistry. In addition, the tortuosity index and vessel lengths were not significantly different (1.05±0.016 and 28.30±3.27 µm in normal tissue, vs. 1.07±0.008 and 26.49±3.18 µm in tumor tissue respectively, p = 0.5357 and p = 0.7033). The daughter/mother ratio (ratio of the sum of the squares of daughter vessel radii over the square of the mother vessel radius) was 1.15±0.09 in normal tissue, and 1.21±0.08 in tumor tissue (p = 0.6531). The confocal laser endomicroscopy is feasible for measuring more vascular parameters from fresh tumor biopsies than conventional immunohistochemistry alone. Provided new contrast agents will be clinically available, future *in vivo* use of CLE could lead to identification of novel biomarkers based on the morphometric characteristics of tumor vasculature.

## Introduction

The imbalance of pro- and anti-angiogenic signaling within tumors creates an abnormal vascular network that is characterized by dilated, tortuous, and hyperpermeable vessels [1], [2]. Therefore the tumor vascularization and in particular the growth of new vessels (angiogenesis) has attracted increased attention in the last two decades for possible applications to diagnosis, prognosis stratification and targeted treatment [1].

Among methods to assess tumor vascularization, the determination of microvessel density (MVD) from immunohistochemistry (IHC) samples is commonly used in preclinical and clinical studies. MVD represents the average number of vessels per mm^2^ within a tissue sample but that measure alone is insufficient for monitoring vascular changes over a treatment period [3]. Other functional and morphologic parameters such as blood volume, permeability, microvessel density, vessel diameter, branching patterns, other measures of vessel shape and tumor metabolism can be associated with tumor angiogenesis.

The mechanisms underlying the enhanced antitumor effects of the combined treatments may differ among the antiangiogenic agents, and have not been determined conclusively. Antiangiogenic agents that disrupt the vascular endothelial growth factor pathway have been demonstrated to normalize tumor vasculature. Some authors consider that the normalized vascular networks are characterized by lower vessel tortuosity and lower vessel density. [4], [5], [6].

The clinical monitoring of the effects of antiangiogenic therapy requires an imaging modality that detects the morphological characteristics of tumor vessels with high sensitivity and specificity. Furthermore, since the antiangiogenic therapy requires repeated treatments, a non-invasive technique is highly desirable [7]. Conventional imaging modalities such as computed tomography, magnetic resonance or ultrasound do not give a detailed image of the tumor vessels, resulting in limited use for *in vivo* cancer evaluation [7]. Non-invasive characterisation of tumour vessels leads to a better understanding of therapy effects and helps to optimise and personalise therapeutic interventions. [8].

Confocal laser endomicroscopy (CLE) allows *in vivo* microscopic analysis of the gastrointestinal mucosa and its microvascularization during endoscopy by using topically or systemically administered contrast agents [7], [9]. Targeting markers of angiogenesis, in association with molecular CLE examinations (immunoendoscopy), adds functional analysis to the morphological aspect of the neoplastic process [7]. Thus, in contrast to the conventional methods of vascular assessment, CLE offers a unique opportunity to selectively enhance different levels of the microvasculature.

In a previous study, we showed that the CLE method is feasible for imaging different aspects of the tumor vasculature from fresh biopsy samples similar to the currently accepted histopathology techniques [9]. In this study, we evaluated new vascular network parameters difficult to assess with conventional immunohistochemistry in patients with clinically staged T3 primary colorectal carcinoma, without metastatic spread, by using CLE combined with fluorescently labeled anti-CD31 antibodies for labeling of both normal and tumor blood vessels.

## Materials and Methods

### Ethics Statement

This study was conducted in accordance to the Declaration of Helsinki and approved by the Ethics Committee of the University of Medicine and Pharmacy of Craiova. A written informed consent was given to each patient prior to study entry.

### Patient Characteristics

The biopsy samples were obtained from five patients recruited at the Research Center of Gastroenterology and Hepatology Craiova, Romania before undergoing any therapy (Table S1). The patients were previously diagnosed with primary colorectal adenocarcinoma (G1–G2 histologic grade) during routine colonoscopy procedures. Patient 1 underwent treatment with bortezomib in combination with chemotherapy and radiation followed by surgery. Patient 2 had a history of surgery followed by chemotherapy for malignant neoplasm of rectosigmoid junction four years before and was diagnosed with local recurrence at follow-up. He was treated with surgery and adjuvant chemotherapy with 5-fluorouracil (5-FU). Patients 3 and 5 underwent surgical resection followed by chemotherapy, with neoadjuvant radiation for the one with rectal carcinoma. Patient 4 had a history of valvular heart disease, chronic hepatitis C infection and low platelet count and was initially treated with FUFOL regimen, but refused further therapy including resection. At the time of this analysis, all five patients were alive at more than one year after the first evaluation.

Immunohistochemistry evaluation was performed on surgical specimens from four patients, while endoscopic biopsies collected before the initiation of chemotherapy were used for the patient who refused the operation.

The patients were prepared for colonoscopy by ingesting a commonly prescribed oral electrolyte lavage solution. Paired biopsies were collected using a colonoscope (CFQ160ZL, Olympus, Tokyo, Japan) from normal mucosa taken at approximately 10 cm from the tumor and from every macroscopically visible lesion and immediately immersed in physiological saline. The fresh biopsy specimens were incubated in the dark with an Alexa-Fluor 488-labeled anti-CD31 (PECAM) antibody (mouse anti-human IgG1, diluted as 1∶10 in PBS with 1% BSA, Exbio, Prague, Czech Republic) for 1 hour at 37°C. The incubation time and dilution ratio were optimized to give the highest penetration inside the tissue and the strongest fluorescence signal for fresh biopsy samples as described before [9]. Immediately after incubation, the biopsy samples were placed on glass slides to be imaged with the CLE method.

### Confocal Laser Endomicroscopy

Imaging was performed using a dedicated endomicroscopy system (Pentax EC-3870 CIFK, Tokyo, Japan), with an excitation wavelength of 488 nm and with a maximum laser power output of ≤1 mW at the surface of the tissue controlled by the user during the examination for optimal imaging contrast. The CLE procedure was performed as follows: the distal tip of the endoscope was placed on the respective mucosal area from the histology glass slides, followed by collection of serial confocal images (7 µm thick, 0.7 µm lateral resolution and the field of view of 475 µm×475 µm) up to a maximum depth of 250 µm by pressing a foot switch pedal.

### CLE Image Processing and Analysis

**Figure 1:**
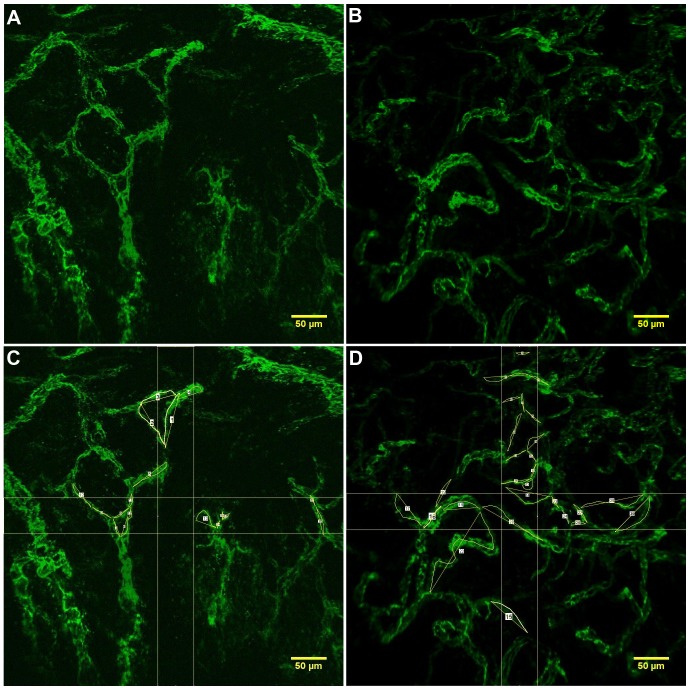
Ex-vivo CLE Vascular measurements of Vessel Length and Tortuosity Index. The Z projection of the image stack for normal (A) and tumor (B) microvasculature, apparently with more tortuous branched vessels. The vessel length was measured using segmented line tool and reported in micrometres. The tortuosity index was calculated as the ratio of segmented and straight line lengths for both normal (C) and malignant (D) human colorectal tissue. *The scale bar is 50 µm.

The location with the best vascular coverage throughout the entire depth of the biopsy sample was chosen for the morphometric measurements. For each patient, 80–100 serial images for each biopsy sample, tumor vs. normal tissue were combined into image stacks. The Z projection of each image stack was obtained using the ImageJ NIH software (National Institutes of Health, Bethesda, Maryland, SUA) to analyze the entire vascular network captured in the biopsy sample (Fig. 1 A and B). All images were calibrated and a color overlay was used for a better distinction of the vasculature. To account for spatial heterogeneity within each sample, two 50×475 µm rectangular regions of interest (ROIs) centered in the middle of each image in the horizontal and vertical direction were marked on the image (Figure 1 C and D). The 50 µm width of the ROI was chosen upon inspection of all samples to allow several bifurcations and entire vessel segments in each sample. The morphometric parameters were measured in each ROI and then averaged to obtain a single value for each sample.

### Vascular Parameter Measurements

The vessel diameters, vessel density, vessel length, tortuosity index and daughter/mother ratio were measured for each vessel in the ROI. The values for the two ROI per patient were averaged to obtain an average value per patient. Finally, the values for each patient were averaged over all patients and the results expressed as the mean ± standard error (Table 1). The vessel density was calculated as the mean number of vessels per ROI divided by the scanned volume (ROI area multiplied by a depth of 250 µm in the Z direction) and then normalized to a 1 mm^3^ volume.

**Table 1:** Average values of the morphometric parameters measured with CLE.

Morphometric Parameters	Normal Mucosa	Colorectal Tumor	P Value
**Vessel Diameter (µm)**	7.60±0.3	9.46±0.4	0.0166
**Vessel Density (vessels/mm^3^)**	3755.79±194.96	5541.05±262.81	0.0006
**Vessel Length (µm)**	28.30±3.27	26.49±3.18	0.7033
**Tortuosity Index**	1.05±0.01	1.07±0.01	0.5357
**Daughter/Mother Ratio**	1.15±0.09	1.21±0.08	0.6531

The values are expressed as the mean ± standard error.

Using the ImageJ’s straight line function, the diameter of each vascular segment which fell entirely or partially in the ROI was measured between two adjacent branching points.

The vessel length for both normal and colorectal cancer tumor samples was measured by tracing the center line of each vessel segment using the segmented line tool. The tortuosity index of each vessel segment was calculated as the ratio of segmented and straight line lengths. The daughter/mother ratio (DMR) was computed as the ratio of the sum of the squares of daughter vessel radii over the square of the mother vessel radius, considering the mother as the largest vessel at a branching point. This measurement is an indication of the departure from a tree structure topology in which case the DMR is roughly one.

### Histopathological Assessment

The histopathological assessment was performed on surgical specimens (rather than the fresh biopsy samples used for CLE), with one exception when an endoscopic biopsy was used from a patient who refused surgical intervention. The specimens were fixed in 4% neutral buffered formalin and processed for routine paraffin embedding and sectioning as 4 µm-thick sections. (Supporting Information S1).

From each block, a slide was stained with hematoxylin- eosin for pathological diagnosis, and the next serial sections were utilized for immunohistochemistry to visualize the CD31 antigen. Briefly, after microwaving in citrate buffer (pH = 6), as an antigen retrieval method, the sections were cooled to room temperature and incubated for 30 minutes in 1% hydrogen peroxide in order to quench endogenous peroxidase. In order to block the unspecific antigenic sites, the specimens were blocked for 30 minutes in 2% skim milk (Bio-rad, München, Germany) and the first antibody was incubated over-night at 4°C (anti-CD31, mouse anti-human, IgG1, clone JC70A, Dako, Glostrup, Denmark, diluted as 1∶100 in PBS with 1% BSA). The next day, the slides were washed, the signal from the first antibody was amplified using a peroxidase-conjugated polymeric system (EnVision Dako, Redox, Bucharest, Romania), and then detected with 3,3′-diaminobenzidine – DAB (Dako). All washing steps were done in 1x PBS buffer. After a hematoxylin staining, the slides were coversliped and evaluated using a Nikon Eclipse 55i microscope (Nikon, Tokyo, Japan) coupled to a 5 Mp color CCD camera (Nikon) and an image-analysis station (Image ProPlus AMS software, Media Cybernetics, Bethesda, Maryland, USA).

Four images from the highest vascular density areas were captured under the same illumination conditions and with a 40x objective for each case, and archived as uncompressed TIFF images. A stylus-design pen was utilized to draw the outlines of the vessels (as visualized by the DAB staining) in the Image ProPlus software package. All these regions of interest (ROIs) were pseudo-colored in black and measured automatically as total vascular areas and total vascular numbers per 40x area, after which the results were normalized for 1 mm^2^ areas. All final measurements utilized the mean values for all the images captured from each case and histopathological profile.

### Statistical Analysis

The morphometric measurements obtained in ImageJ were exported for data analysis in Microsoft Office Excel (Microsoft, Redmond, Washington, USA). Results were expressed as the mean ± standard error. All measured vessel parameters were considered to be derived from a normal distribution as before [9], [10], and therefore statistical differences between normal and tumor vasculature were calculated using the Student’s t-test, and p-values ≤0.05 were considered to be statistically significant.

## Results

### Confocal Laser Endomicroscopy

The CLE procedure gave a detailed observation of adjacent tumor and normal mucosa sections with visible fluorescent signal (Fig. 1). Both the normal and tumor vessels seem tortuous overall but it was not clear from the visual analysis of the images whether there is a significant difference between normal and tumor vessels or whether the tortuosity is maintained at the level of individual vascular fragments.

The mean diameter of vessels in the tumor samples (9.46±0.4 µm) was significantly larger than in the normal sample (7.60±0.3 µm, p = 0.0166) (Table 1). Similarly, the vessel density was significantly higher in the colorectal cancer (5541.05±262.81 vessels/mm^3^) than the normal colorectal mucosa (3755.79±194.96 vessels/mm^3^, p = 0.0006).

Contrary to the visual evaluation, the tortuosity index and vessel length were not statistical different between normal (tortuosity index was 1.05±0.016 and vessel length was 28.30±3.27 µm) and tumor tissue (tortuosity index was 1.07±0.008 and vessel length 26.49±3.18 µm) (Table 1). The mean daughter/mother ratio was 1.15±0.09 in the normal sample and 1.21±0.08 in the tumor samples (p = 0.6531). The data for each patient are presented in Table 2. Although a small sample size, there are no large variations between patients. As a possible exception, in patient 2 the values for the daughter/mother ratios were larger than 1 in both the tumor and normal samples and (1.53 and 1.46 respectively) which might reflect a particularly vascularized and heterogeneous vasculature but on average over all patients, the values were close to 1.

**Table 2:** Vascular parameters of individual patients measured with the CLE method.

Confocal Laser Endomicroscopy						
Patient	Control			Tumor		
	DMR	VL	TI	DMR	VL	TI
**1**	1.22	36.27	1.03	1.14	26.67	1.06
**2**	1.46	25.18	1.06	1.53	25.70	1.07
**3**	1.17	21.48	1.12	1.07	15.24	1.10
**4**	0.86	22.48	1.05	1.20	30.81	1.07
**5**	1.05	36.10	1.03	1.12	34.07	1.05

DMR- daughter/mother ratio, VL- vessel length, TI- tortuosity index.

### Immunohistochemistry

In order to see whether the CLE vessel signals were correlated with histological data, the vessel diameter and the vessel density detected by CLE were qualitatively compared to the CD31-stained vessels in a histological section from the same patient. The vascular area was 8.19±1.35% in colorectal cancer, significantly higher than in normal mucosa, 3.17±0.48% (p = 0.0084). The MVD for the colorectal normal mucosa and tumor tissue were also significantly different (185.73±33.21/mm^2^ and 309.28±32.26/mm^2^ respectively, p = 0.0280, Table 3).

**Table 3:** Average vascular parameters measured from Anti-CD31 Immunohistochemistry samples.

Vascular Parameters	Normal Mucosa	Colorectal Tumor	P Value
**Vascular Area (%)**	3.17±0.48%	8.19±1.35%	0.0084
**MVD (vessels/mm^2^)**	185.73±33.21	309.28±32.26	0.0280

The values are expressed as the mean ± standard error.

## Discussions and Conclusions

Typically, in tumor vascular studies the microvasculature is described only from the vessel diameter and density points of view because these features can be measured from histological slices. We have also performed an immunohistochemical study on our samples, and obtained similar results to the literature (larger MVD and vascular area in tumors vs. normal tissue) [9], [11], [12], [13]. In addition, we evaluated more parameters of vascularization from volumetric samples, such as the vessel length, the index of tortuosity and the daughter/mother ratio, that can be easily quantified with CLE due to its greater penetration depth into the tissue (up to 250 µm). To our knowledge, this is the first report of the daughter/mother ratios and tortuosity index in colorectal cancer vasculature.

Anatomically, tumor microvessels are generally more dilated, tortuous, and saccular with irregular patterns of interconnection and branching [14]. The overexpression of some pro-angiogenic factors, like VEGF, leads to formation of a new vasculature that is structurally abnormal at macroscopic and microscopic levels and it is generally considered that these abnormalities are exacerbated as the tumor continues to develop [15]. Consequently, the microvessel density increases gradually from low-grade to high-grade neoplasia to cancer [16]. Similarly in the present study we have found a significantly higher vessel density and diameter of tumor vessels.

Most of the literature gives only a qualitative analysis on the vessel length and tortuosity in colorectal cancer. Yuan et al. (1996) provided early evidence that neutralization of tumor-cell-derived VEGF could reverse some of the abnormalities of the tumor microvasculature like vascular diameter and tortuosity [17]. Taking into consideration the fact that one strategy for successful treatment with anti-angiogenic agents is to reduce the tortuosity of the abnormal vessels [18], a quantitative assessment is necessary. Generally, vascular tortuosity was evaluated from 2D images [19]. Previous research defined different types of vessel tortuosity depending on vessel length and showed that vessel tortuosity and length are variable in tumors [20]. In our confocal optical sections, the malignant blood vessels appeared more dilated and more tortuous compared to the normal samples. Interestingly, in spite of the visual examination, we obtained no statistical significance when we calculated their index of tortuosity. This could reflect the fact that the tortuous appearance might be given by the mechanical deformation as the entire tumor tissue grows rather individual vessel fragments being tortuous. One recent three- dimensional quantification study showed no differences regarding tortuosity and vessel length between capillaries in healthy and cancerous tissues [21], while quantitative studies in three-dimensional tumors at later stages of growth showed a decrease in vessel length [20], [22], [23].

There was not a significant difference in the daughter/mother ratio between normal and tumor branching network and the values are close to 1 (1.15±0.09 vs. 1.21±0.08). This parameter is derived from Murray’s law for connecting large vessels to small vessels applied in colorectal cancer angiogenesis. Murray’s law, available for asymmetrical as well as symmetrical branching systems, states that the cube of the radius of a parent vessel equals the sum of the cubes of the radii of the daughters and refers to the optimization of blood vessel diameter and of blood volume according to the metabolic requirements [24]. One limitation of our study is that the direction of blood flow is not known, therefore the exact designation of the mother vessel cannot be specified. This shortcoming could be addressed with the *in vivo* observation of blood flow in tumors, using clinically approved contrast agents.

The utility of using morphometric parameters as biomarkers could depend on the stage of tumor development. We calculated the morphometric parameters which measure different aspects of colorectal tumor microvasculature taking into consideration only T3 staged, well to moderately differentiated carcinoma (G1–G2 grade), without metastasis. In 1997, Robert D. Cardiff et al. calculated the index of tortuosity in a study based on a mouse mammary tumor model system, using histology combined with computer-assisted intravital microscopy and mice injected with FITC [25]. He considered this parameter a prognostic factor and demonstrated that it was statistically significant when correlated with a higher metastatic rate. Similarly, further long-term studies on larger groups of patients are necessary to see if all morphometric parameters show strong association with colorectal adenocarcinoma grading and if they might correlate with treatment outcome and prognosis.

Other methodology could be applied to studies which monitor the effect of anti-angiogenesis therapy (e.g. bevacizumab) on vascular branching, tortuosity and vessel volume in tumors [8], [26].

As the efficacy of chemotherapy depends on the presence of an adequate tumor blood supply to ensure drug delivery, “normalization” of the tumor vascular network is desirable. Thus, assessing the differences between the vessel length, tortuosity and branching patterns with CLE imaging and fresh biopsy staining could be useful for identifying the cathegory of patients who might benefit from neoadjuvant angiogenetic therapy. [27].

Currently, three-dimensional confocal laser endomicroscopy reconstruction represents a challenge [28]–[30]. Using CD 31 antibodies fluorescently labeled with a fluorochrome (Alexa Fluor 488) and CLE for identification of vessels from fresh biopsy samples, we could estimate their shape, branching features and density. Two major advantages of this method are the shorter processing time (an hour vs. several hours to days), and no artifacts from tissue fixation and processing for IHC. Future similar studies could include more specific markers for tumour neoangiogenesis as compared to the panendothelial marker used in the current study.

A direct comparison of the morphometric parameters between CLE and IHC is difficult and of limited relevance. One would have to compare the results from one paraffin-embedded tissue section used in conventional IHC for clinical diagnosis, with the information from the 3D stack obtained in a single CLE scan that was analyzed in the present study. A 2D slice does not allow an accurate measurement of the vessel length, tortuosity or mother/daughter ratios. Another difficulty is that the CD31 antibody and the tissue processing steps (fresh versus fixed and paraffin-embedded) are different between the two staining techniques, and are likely to generate different values for the same parameter due to differences in processing techniques.

In summary, in the current manuscript we demonstrated that the confocal laser endomicroscopy is feasible for measuring more vascular parameters from fresh tumor biopsies than conventional immunohistochemistry alone. Moreover, we have presented a framework that captures some of the essential features of colorectal cancer microvascularization, using a reproducible non-invasive technique and targeted panendothelial markers on fresh biopsies. Verification should be done in future experiments using confocal laser endomicroscopy *in vivo* by manipulating tumor morphology through variations in tumor vascularization. Provided new contrast agents will be clinically available, future *in vivo* use of CLE could lead to identification of novel biomarkers based on the morphometric characteristics of tumor vasculature.

The results are useful for further understanding of the colorectal cancer development and patient selection criteria. Furthermore, as defined by Ehling J et al (2013), non-invasive characterisation of tumour vessels leads to a better understanding of therapy effects, helps optimize and personalize therapeutic interventions [8]. The results extend our previous feasibility findings to the evaluation of new vascular parameters, which are important in the management of cancer, but which are difficult to measure from immunohistochemical sections (the vessels/mm^3^, the vessel length, the index of tortuosity and the mother/daughter ratio). We have shown the reproducibility of the method by comparing the two common used parameters (vessel diameter, vessel density) to the traditional immunohistochemistry. Using CLE imaging technique for analyzing the 3D microvascular architecture of tumors is suitable for both quantitative and qualitative angiogenesis measurements in vivo and combined with targeted antibodies.

## Supporting Information

Table S1 **Patient characteristics and immunohistochemistry results.**
(DOC)
