# Supplementary material for: Evaluation of New Morphometric Parameters of Neoangiogenesis in Human Colorectal Cancer Using Confocal Laser Endomicroscopy (CLE) and Targeted Panendothelial Markers
Source: PLoS One. 2014 Mar 10;9(3):e91084. doi: 10.1371/journal.pone.0091084 (PMC3948726; doi:10.1371/journal.pone.0091084)
Supplement: Table S1 — Patient characteristics and immunohistochemistry results. (DOC) [file pone.0091084.s001.doc]

**Table S1. Patient characteristics and immunohistochemistry results**

| **Patient Characteristics** | | | | | | | | | | **Immunohistochemistry** | | | |
| --- | --- | --- | --- | --- | --- | --- | --- | --- | --- | --- | --- | --- | --- |
|  |  | | | | | | | | | **Control** | | **Tumor** | |
| **Patient** | **Gender** | **Age** | **Tumor Location** | | **Tissue Source** | **Tumor Grading** | **Preoperative Stage** | **Rht** | **Cht** | **Vascular Area (%)** | **MVD** | **Vascular Area (%)** | **MVD** |
| **1** | M | 54 | Rectum | Surgery | | G1/G2 | T3N1M0 | + | Neoadj | 2.27 | 93.06 | 6.57 | 191.59 |
| **2** | M | 49 | Rectosigmoid junction | Surgery | | G1/G2 | T3N0M0 | - | Adj | 2.19 | 205.28 | 8.55 | 301.07 |
| **3** | M | 78 | Sigmoid colon | Surgery | | G2 | T3N0M0 | - | Adj | 3.89 | 145.88 | 8.02 | 383.18 |
| **4** | M | 75 | Rectum | Biopsy | | G2 | T3N0M0 | - | Neoadj | 2.84 | 292.86 | 12.96 | 342.13 |
| **5** | M | 60 | Rectum | Surgery | | G2 | T3N0M0 | + | Adj | 4.70 | 191.59 | 4.86 | 328.44 |

Abbreviations: Rht- radiotherapy, Cht- chemotherapy, Neoadj- Neoadjuvant chemotherapy, Adj- Adjuvant chemotherapy, MVD- microvessel density (vessels/mm^2^)
